# A Novel Two-Wire Fast Readout Approach for Suppressing Cable Crosstalk in a Tactile Resistive Sensor Array

**DOI:** 10.3390/s16050720

**Published:** 2016-05-18

**Authors:** Jianfeng Wu, Yu Wang, Jianqing Li, Aiguo Song

**Affiliations:** School of Instrument Science and Engineering, Southeast University, Nanjing 210096, China; 220152746@seu.edu.cn (Y.W.); ljq@seu.edu.cn (J.L.); a.g.song@seu.edu.cn (A.S.)

**Keywords:** resistive sensor array, measurement error, full 2-wire fast readout approach

## Abstract

For suppressing the crosstalk problem due to wire resistances and contacted resistances of the long flexible cables in tactile sensing systems, we present a novel two-wire fast readout approach for the two-dimensional resistive sensor array in shared row-column fashion. In the approach, two wires are used for every driving electrode and every sampling electrode in the resistive sensor array. The approach with a high readout rate, though it requires a large number of wires and many sampling channels, solves the cable crosstalk problem. We also verified the approach’s performance with Multisim simulations and actual experiments.

## 1. Introduction

Resistive sensor arrays are used in many applications such as tactile sensing [1,2,3,4,5,6], temperature sensing [6], infrared sensing [7], *etc.* With resistive sensor arrays, wearable sensors such as wearable Braille sensors [8], electronic skin devices [5,6,9], and smart clothes [10] have been proved to be useful for monitoring the activities of humans [5,6,9,10,11,12] and robots [1,2,3,4,13]. For comfort wearing and precisely perceiving the physical parameter distribution of the sensing domain, interface electronics should not change the flexibility near the tactile resistive sensor array in smart clothes, and a readout circuit with long flexible cables would be a good choice to read out all elements in the array. Readout circuits of resistive sensor arrays with long flexible cables are also useful for robotic applications with limited space on their robot structures, for example the gripper [5]. Thus, in tactile resistive sensor arrays, a large number of sensors and long flexible cables were necessary for sensing with better spatial resolution and flexibility in robotic operations [1,2,3,4,13] and electronic skin devices [5,6,9,10,11,12]; however, these increased the interconnect complexity and degraded the performance of measurement accuracy in their readout circuits. Unfortunately, it is still a problem for the resistive sensor array with long cables to have a readout approach with good accuracy and a high readout rate.

In this paper, we propose a novel two-wire approach based on the zero potential method (ZPM) with a high readout rate for suppressing the cable crosstalk problem. It uses 2(*M* + *N*) wires, (*M* + *N*) op-amps in negative feedback, and 2*M* synchronous sampling channels in the resistive sensor array with *M* × *N* elements, keeping all sampling electrodes and all driving electrodes virtually at equipotential and reducing the cable crosstalk sufficiently.

## 2. Theoretical Analysis

Many readout approaches have been propose to suppress crosstalk in the tactile resistive sensor array. With detailed theoretical analysis and experimental results, a novel two-wire readout circuit with high accuracy, low cost and simple structure was presented in [14]. The measurement error of the element being tested (EBT) in the resistive sensor array with long cables was affected by many factors such as parasitic parallel currents of the non-scanned elements [14,15], switch-on resistances of the multiplexers [14], wire resistances of the long cables [16,17], and contact resistances between the tested cables’ plugs and the test circuits’ sockets [16,17]. The effect of parasitic parallel currents of the non-scanned elements would be more significant with a larger array size in the resistive sensor array. Different multiplexers had various switch-on resistances (*R_sc_*s) which had resistances of several hundred milliohms to several hundred ohms [16] and varied with the change of supply voltages. With the increase of the cable’s length, for example 500 mm [5], wire resistances would increase. With the change of mechanical vibration and time, contact resistances would vary in the range of tens of milliohms to several ohms [16,17].

Many readout approaches were proposed to reduce the measurement error of the EBT caused by these factors. Table 1 showed the comparison of different readout approaches. With the minimum circuitry of one sampling op-amp, the crosstalk caused by the non-scanned elements in the resistive sensor array was reduced with the readout methods including the voltage feedback method (VFM) [18,19] and ZPM [15]. Also, the measurement error caused by the multiplexers’ *R_sc_*s was reduced by the readout methods based on VFM [18,19]. With the two-wire VFM [16] and the two-wire setting non-scanned-driving-electrode equipotential (S-NSDE-EP) method [17], the crosstalk caused by wire resistances and contact resistances was well suppressed by using two wires and one op-amp in negative feedback for every driving electrode and every sampling electrode. However, these methods could access only one sensitive element in the resistive sensor array at the same time, which caused their low readout rates. Based on VFM with one op-amp and one sampling channel, Speeter [4] realized a 16 × 16 tactile sensing system for robotic manipulation with a scanning frame rate of 60 Hz.

**Figure 1:**
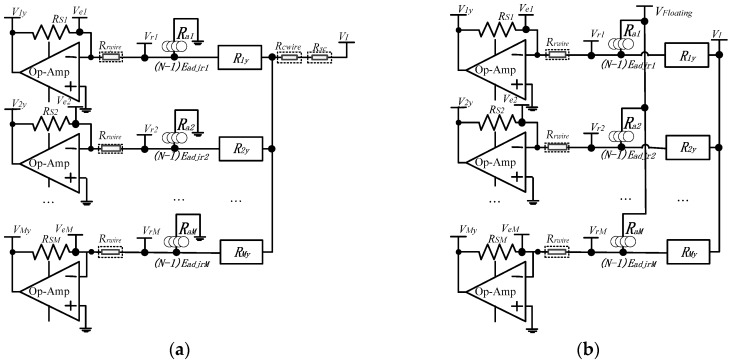
(**a**) Equivalent circuit model of the multi-channel one-wire readout circuit (Model A); (**b**) Equivalent circuit model of the multi-channel part two-wire readout circuit (Model B).

Fast readout approaches of resistive sensor arrays made it possible for the robot to quickly respond to an external stimulus. With many synchronous sampling channels and many op-amps in negative feedback to access all elements on one column or one row at the same time, fast readout methods including the ZPM [5,6,13,20], the passive integrator method (PIM) [1,2], and the resistance matrix approach (RMA) [12] were proposed without inserting additional component in the tactile resistive sensor array. Using the ZPM with many sampling channels and many op-amps in negative feedback, Yang *et al.* [6] realized a 32 × 32 temperature and tactile sensing array with a maximum scanning rate greater than 3000 elements per second. Luo *et al.* [13] realized a 16 × 16 flexible resistive sensor array with a scanning frame rate of 1.2 kHz. With high readout rates for using many sampling channels, the crosstalk caused by the adjacent elements in the resistive sensor array was suppressed by the ZPM-based one-wire readout circuit [5,6,13,20] (the one-wire readout circuit hereafter) with its equivalent circuit model (Model A) as shown in Figure 1a, using only one wire for both every driving electrode and every sampling electrode; the crosstalk caused by the long cables was partly suppressed by the ZPM-based part two-wire readout circuit [14] (the part two-wire readout circuit hereafter) with its equivalent circuit model (Model B) as shown in Figure 1b, using two wires for every driving electrode but only one wire for every sampling electrode. In Model A and Model B, all elements on one column or one row could be accessed at the same time by many synchronous sampling channels and fast readout rates were obtained. As shown in Model A, the equivalent effect adjacent resistance (*R_ax_,* *x* = 1 to *M*) of (*N* − 1) row-adjacent non-scanned elements of every element (*R_xy_*, *x* = 1 to *M*, *y* = 1 to *N*) on the scanning column was connected to zero potential and its bypass effect was eliminated by ZPM. As shown in Model B, with the equivalent effect adjacent resistance (*R_ax_,* *x* = 1 to *M*), (*N* − 1) row-adjacent non-scanned elements of every element (*R_xy_*, *x* = 1 to *M*, *y* = 1 to *N*) on the scanning column were connected to the non-scanning column electrodes with uncertain floating potentials.

In Model A, the multiplexer’s switch-on resistance (*R_sc_*) and the column resistance (*R_cwire_*) including wire resistance and contact resistance existed on the scanning column electrode. Therefore, the voltage on the scanning column electrode was not equal to the set voltage (*V_I_*) and the crosstalk caused by multiplexers and column wires existed in the one-wire readout circuit. In Model B, the voltage on the scanning column electrode was equal to *V_I_* by using two wires and one op-amp in negative feedback for every driving electrode and then the crosstalk caused by multiplexers and column wires was suppressed by the part two-wire circuit. In Model A and Model B, with the row resistance (*R_r__wire_*) including wire resistance and contact resistance, only one wire was used for every sampling electrode. Thus, the voltage on every row electrode in the resistive sensor array was not at zero potential and the crosstalk caused by row wires still existed in these two circuits. Based on the ZPM with a larger number of sampling op-amps with a fast scanning rate, we proposed the full two-wire readout circuit to suppress the crosstalk caused by both the column resistances and the row resistances.

**Figure 2:**
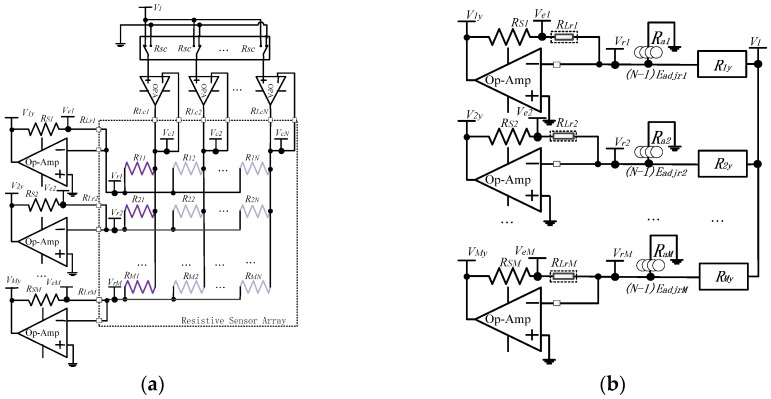
(**a**) Full two-wire readout circuit; (**b**) Equivalent circuit model of the full two-wire readout circuit (Model C).

Figure 2 shows the proposed full two-wire readout circuit and its equivalent circuit model, in which two wires and one identical op-amp in negative feedback were used for both every sampling electrode and every driving electrode, where sampling electrodes and driving electrodes were row electrodes and column electrodes, respectively. As shown in Figure 2a, all elements on the first column in the array were selected and accessed. On the column electrodes, *N* 2:1 multiplexers had switch-on resistances (*R_sc_*s). On all electrodes in the resistive sensor array, each column wire had the column resistance (*R_Lc__y_*, *y* = 1 to *N*) and each row wire had the row resistance (*R_Lrx_*, *x* = 1 to *M*), with *R_Lc__y_* and *R_Lrx_* having wire resistance and contact resistance. The virtual equipotential appearing at the two inputs of the op-amp on every driving electrode kept the scanning column electrode at the set voltage (*V_I_*) and all other column electrodes at zero potential. The virtual equipotential appearing at the two inputs of the op-amp on every sampling electrode also kept each row electrode at zero potential, and the sampling electrode’s voltage (*V_rx_*, *x* = 1 to *M*) was equal to zero potential in the full two-wire readout circuit. Therefore, with the full two-wire readout circuit, two terminals of every non-scanned element had equal potential and no bypass current existed on all non-scanned elements. As shown in Model C, the bypass effect of the equivalent effect adjacent resistance (*R_ax_,* *x* = 1 to *M*) of (*N* − 1) row-adjacent non-scanned elements (*R_non-scanned_*s) of every element (*V_xy_*, *x* = 1 to *M*, *y =* 1 to *N*) on the scanning column were eliminated. Thus, the crosstalk caused by multiplexers’ *R_sc_*s and column resistances of the column wires was suppressed.

When *V_I_* was applied to the scanning column electrode on the *y*th column (*y* = 1 to *N*), the current on the EBT (*R_xy_*, *x* = 1 to *M*, *y* = 1 to *N*) of the *x*th row was *V_I_*/*R_xy_*. These currents would flow through each feedback resistance (*R_Sx_*, *x* = 1 to *M*) of all the *M* row op-amps, producing an output voltage (*V_xy_*, *x* = 1 to *M*, *y* = 1 to *N*) equal to Equation (1).

*V_xy_* = − (*R_Sx_ + R_Lrx_*) × *V_I_*/*R_xy_* (1)

Thus, we could read *M* elements on one column at the same time. However, as shown in Figure 2, the *R_Lrx_* caused the difference between the sampling electrode’s voltage (*V_r__x_* = 0, *x* = 1 to *M*) and the voltage (*V_ex_*, *x* = 1 to *M*) on the shared node of *R_Lrx_* and *R_S__x_*. Also, the row resistance would vary with both the change of the wire length and the change of the contact state for mechanical vibration and time variation. So the row resistance still affected the EBT’s measurement error.

As the currents on *R_xy_*, *R_Lr__x_*, and *R_Sx_* were equal, *R_Sx_* were known, and *V_xy_* and *V_ex_* could be measured by two synchronous sampling channels in the analog digital converter (ADC). So the equivalent resistance value (*R_xy_*) of the EBT in the full two-wire circuit could be calculated with Equation (2).

*R_xy_* = *V_I_* × *R_Sx_*/(*V_ex_* − *V_xy_*) (2)

No *R_Lr__x_* existed in Equation (2). Thus, the crosstalk caused by row resistances of row wires was also suppressed in the full two-wire readout circuit.

From the above discussion, with 2*M* synchronous sampling channels, the proposed full two-wire readout approach can read *M* elements on the same column at the same time and suppress the crosstalk caused by multiplexers and cables completely.

## 3. Experiments

### 3.1. Simulation Experiments

To evaluate the performances of the proposed full two-wire readout circuit, a precise op-amp, OP07, was selected as the macro-model of the op-amp in the simulations of National Instrument (NI) Multisim 12. The resistance value of the force sensing resistor (One-inch ShuntMode FSR of Sensitronics) was in the range of 12 kΩ to 100 kΩ for force in 240–4000 grams [21] and the resistance value of each thermistor (MF52B-103F950 thermistor of the SinoChip Electronic Co., Ltd. Nanjing, China) in the temperature tactile device was in the range of 3.6 kΩ to 20 kΩ for temperature in the range of 10–50 °C [22]. Therefore, in simulation experiments, all elements on the scanning column, which could be sampled at the same time in three readout circuits, including the one-wire readout circuit [20], the part two-wire readout circuit [14], and the proposed full two-wire readout circuit, were set at 1 kΩ, 2 kΩ, 4 kΩ, 8 kΩ, 16 kΩ, 32 kΩ, 64 kΩ, and 128 kΩ, respectively; *V_I_* was set at −5 V, all *R_Sx_*s were set at 1 kΩ, and the positive and negative voltage sources of the op-amps were set at 9 V and −9 V, respectively.

#### 3.1.1. Experiments of the Effect of the Column Resistance and the Row Resistance

**Figure 3:**
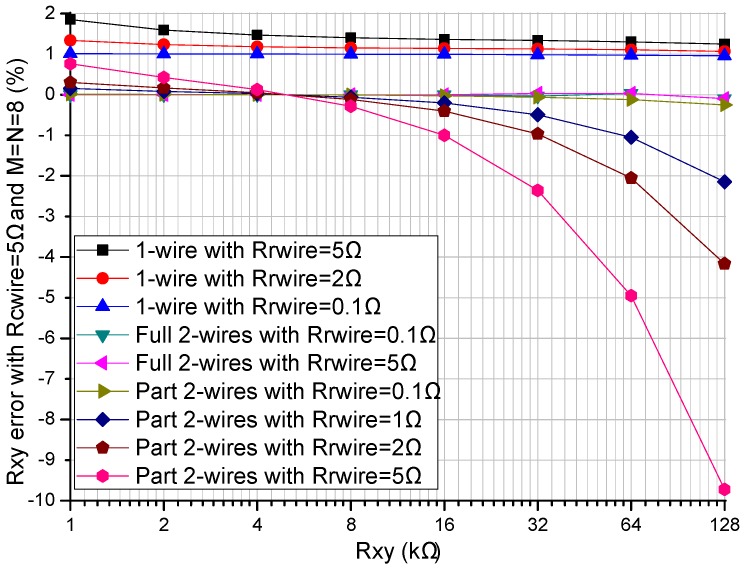
Effect of the *R_rwire_* on the *R_xy_* errors in the three circuits where *R_cwire_* = 5 Ω and *M* = *N* = 8.

**Figure 4:**
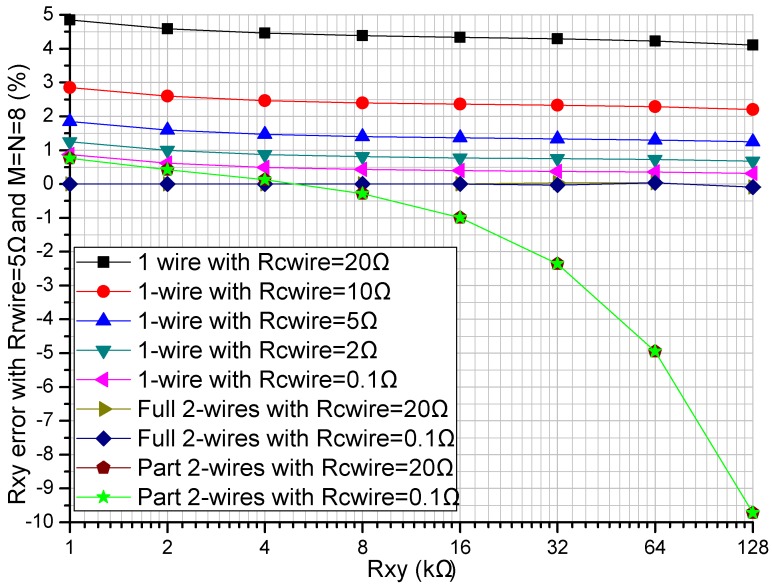
Effect of the *R_cwire_* on the *R_xy_* errors in the three circuits where *R_rwire_* = 5 Ω and *M* = *N* = 8.

The readout circuit’s performance in the resistive sensor array was affected by the row resistance (*R_Lrx_ = R_rwire_*, *x* = 1 to *M*) and the column resistance (*R_Lcy_ = R_cwire_*, *y* = 1 to *N*). The *R_rwire_* including row wire resistance and row contact resistance could vary from tens of milliohms to several ohms [16,17]. The *R_cwire_* including the multiplexers’ switch-on resistance, column wire resistance, and column contact resistance could vary from several hundred milliohms to several hundred ohms [16]. The effect of the multiplexers’ switch-on resistance could be reduced by using the multiplexers with a small switch-on resistance, for example 0.41 Ω in ADG884. We investigated the effect of the *R_rwire_* and the *R_cwire_* on three readout circuits including the one-wire readout circuit [20], the part two-wire readout circuit [14], and the proposed full two-wire readout circuit in NI Multisim. In simulations, we fixed some parameters including all non-scanning elements in the resistive sensor array at 10 kΩ, and *M* and *N* at 8. In the experiment of the row resistance’s effect, all *R_cwire_*s were set at 5 Ω and all *R_rwire_*s at the same resistance varied from 0.1 Ω to 5 Ω. In the experiment of the column resistance’s effect, all *R_rwire_*s were set at 5 Ω and all *R_cwire_*s at the same resistance varied from 0.1 Ω to 20 Ω. The results of the experiments are shown in Figure 3 and Figure 4.

From Figure 3, with all *R_cwire_*s fixed at 5 Ω, all *R_rwire_*s at the same resistance varied from 0.1 Ω to 5 Ω, *R_xy_* varied from 1 kΩ to 128 kΩ, and *R_xy_* errors in the full two-wire readout circuit showed a tiny variation (less than 0.1%), while *R_xy_* errors in the one-wire readout circuit and the part two-wire readout circuit showed obvious changes. With the increase of the resistance of *R_xy_*, both the one-wire readout circuit and the part two-wire readout circuit showed *R_xy_* errors with negative coefficients, which were more significant in the part two-wire readout circuit with a larger resistance of *R_rwire_*. When *R_rwire_* was small (less than 0.1 Ω), the *R_xy_* errors of the part two-wire readout circuit were small even if *R_cwire_* was large.

From Figure 4, with *R_rwire_* fixed at 5 Ω, *R_xy_* errors of the one-wire readout circuit showed obvious changes for variations of both *R_xy_* and *R_cwire_*; *R_xy_* errors of the part two-wire readout circuit showed tiny variations for resistance variations of *R_cwire_* but obvious changes for resistance variations of *R_xy_*, which were caused by the large resistance of *R_rwire_*. With the *R_cwire_* varied from 0.1 Ω to 20 Ω and *R_xy_* varied from 1 kΩ to 128 kΩ, *R_xy_* errors in the full two-wire readout circuit showed tiny variations.

The proposed full two-wire readout circuit had a better performance than the one-wire readout circuit and the part two-wire readout circuit when *R_rwire_* was varied from 0.1 Ω to 5 Ω and *R_cwire_* was varied from 0.1 Ω to 20 Ω; the absolute *R_xy_* errors in the full two-wire circuit were small enough to be negligible (less than 0.1%) when *R_rwire_* was less than 5 Ω and *R_cwire_* was less than 20 Ω. Column resistance had an obvious effect on the *R_xy_* errors of the one-wire readout circuit, but it had an insignificant effect on the *R_xy_* errors of both the part two-wire readout circuit and the full two-wire readout circuit. Row resistance had an obvious effect on the *R_xy_* errors of both the one-wire readout circuit and the part two-wire readout circuit, but it had a negligible effect on the *R_xy_* errors of the full two-wire readout circuit.

#### 3.1.2. Experiment of the Non-Scanned Elements’ Effect

**Figure 5:**
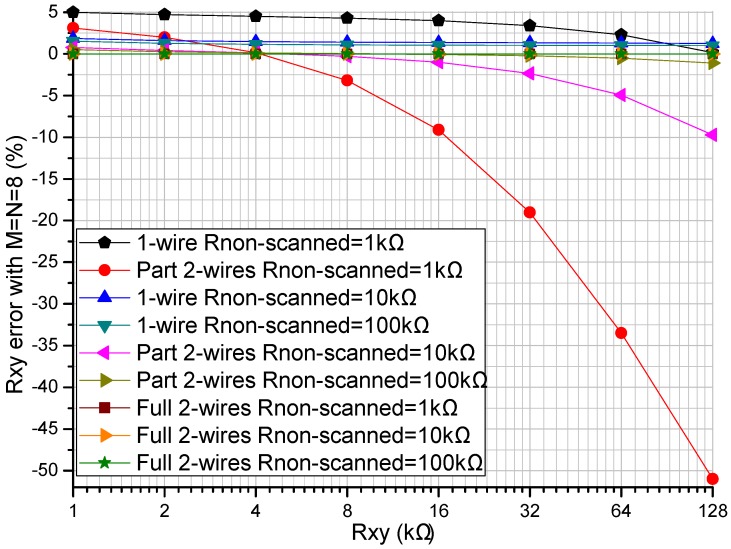
Effect of *R_non-scanned_* on the *R_xy_* errors in the three circuits where *R_rwire_* = *R_cwire_* = 5 Ω and *M* = *N* = 8.

The performance of the two-dimensional (2D) resistive sensor arrays was affected by the non-scanned elements (*R_non-scanned_*s) [14,15]. We investigated the effect of *R_non-scanned_*s on three readout circuits including the one-wire readout circuit [20], the part two-wire readout circuit [14], and the proposed full two-wire readout circuit in NI Multisim. In simulations, we fixed some parameters including the *R_rwire_* and the *R_cwire_* at 5 Ω, and *M* and *N* at 8. All *R_non-scanned_*s of the three readout circuits had the same resistance of 1 kΩ, 10 kΩ, or 100 kΩ, and the simulation results are shown in Figure 5.

From Figure 5, with *R_rwire_* and *R_cwire_* fixed at 5 Ω, all *R_non-scanned_*s of the three readout circuits at the same resistance of 1 kΩ, 10 kΩ, or 100 kΩ, and *R_xy_* varied from 1 kΩ to 128 kΩ, *R_xy_* errors in the full two-wire readout circuit showed a tiny variation (less than 0.1%), while *R_xy_* errors in the one-wire readout circuit and the part two-wire readout circuit showed significant changes with obvious negative coefficients, which were more significant for *R_non-scanned_*s with smaller resistance. Thus, with *R_0_* fixed at 5 Ω, all *R_non-scanned_*s varied from 1 kΩ to 100 kΩ and *R_xy_* varied from 1 kΩ to 128 kΩ, the *R_xy_* errors in the full two-wire readout circuit were small enough to be negligible (less than 0.1%) and the full two-wire readout circuit had a better performance than the one-wire readout circuit and the part two-wire readout circuit.

#### 3.1.3. Experiment of the Column Number’s Effect

**Figure 6:**
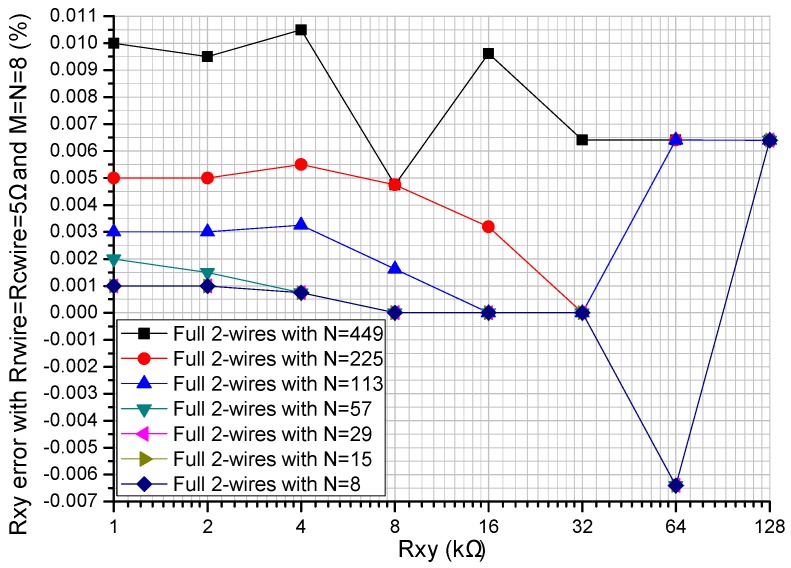
Effect of *N* on the *R_xy_* errors in the full two-wire circuit where *R_rwire_* = *R_cwire_* = 5 Ω and *M* = *N* = 8.

Array size, such as the row number (*M*) and the column number (*N*), was proved to affect the performance of the *M* × *N* resistive sensor arrays [14,15,16,17]. The good performance of the part two-wire readout circuit and the bad performance of the one-wire readout circuit in suppressing the crosstalk caused by *M* and *N* were presented in [14]. From Figure 2, the full two-wire readout circuit needed 2*M* synchronous sampling channels, which were difficult to realize for the array with a large row number. With a larger column number, the full two-wire readout circuit could access more elements. In this experiment, we investigated the effect of *N* on the full two-wire readout circuit in NI Multisim. In simulations, we fixed some parameters including *R_rwire_* and *R_cwire_* at 5 Ω, all non-scanning elements in the resistive sensor array at 10 kΩ, and *M* at 8. *N* varied from 8 to 449, and the results are shown in Figure 6.

From Figure 6, with *R_xy_* in the range from 1 kΩ to 128 kΩ, with the increase of *N* from 8 to 449, *R_xy_* errors in the full two-wire readout circuit increased within a small range from −0.007% to 0.011%; with *N* in the range from 8 to 449, with the increase of *R_xy_* from 1 kΩ to 128 kΩ, *R_xy_* errors in the full two-wire readout circuit showed tiny non-monotone variations, which were caused by the sampling-quantization error in the digital measurement. Thus, the full two-wire readout circuit was applicable for the resistive sensor array with a large column number.

### 3.2. The R_xy_ Measurement Experiment with the Full Two-Wire Fast Readout Prototype Circuit

A prototype circuit based on the full two-wire fast readout approach was designed. In the prototype circuit, OPA4209 (from the datasheet, the offset voltage, the bias current, the gain-bandwidth, and the gain are equal to ±150 μV, ±1 nA, 18 MHz, and 120 dB, respectively) was used as the op-amp, CD4053 (from the datasheet, the on-resistance and the on-resistance match between channels are equal to 150 Ω and 5 Ω, respectively) was used as the multiplex switch, and the resistances of the sampling resistors were set at 4.7 kΩ. In the prototype circuit, *M* and *N* were 4 and 6, respectively, and 20 lines were necessary for the prototype circuit. A cable with a length of 400 mm, which had wire resistances from 0.082 Ω to 0.179 Ω in a pre-experiment, was used to connect the resistive sensor array modules with the circuit. In the prototype circuit, *V_I_* was −4.980 V and the AD2738 with eight channels and a 12 bit ADC were used.

**Figure 7:**
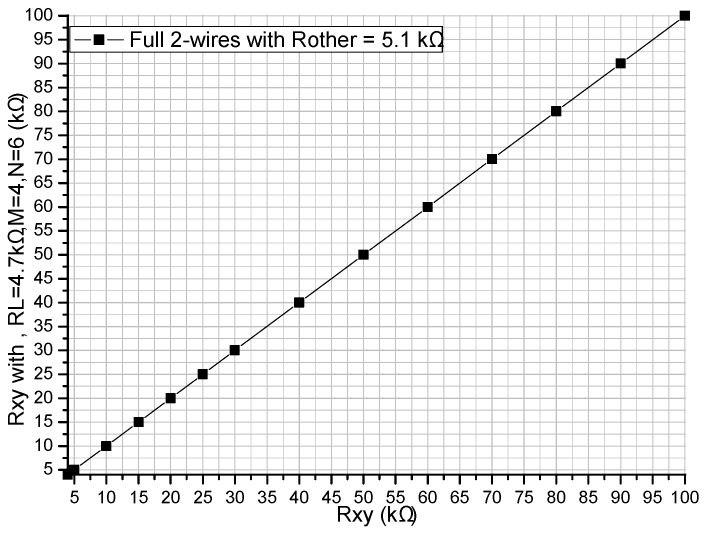
Measurement result of the EBT’s resistance in the full two-wire prototype circuit.

In the experiment, one EBT was replaced by a precision resistance box with its smallest step resistance value at 0.1 Ω and all other elements were precision resistors at 5.1 kΩ. The resistance value of the EBT was varied from 4 kΩ to 100 kΩ, and the resistances in the full two-wire fast readout prototype circuit are shown in Figure 7. In this experiment, *V_xy_* and *V_ex_* were measured by two sampling channels in the ADC, and the resistances of the EBT as shown in Figure 7 were calculated with Equation (2).

From the results in Figure 7, we found that the full two-wire fast readout prototype circuit had a good performance in a wide range of the elements being tested.

**Figure 8:**
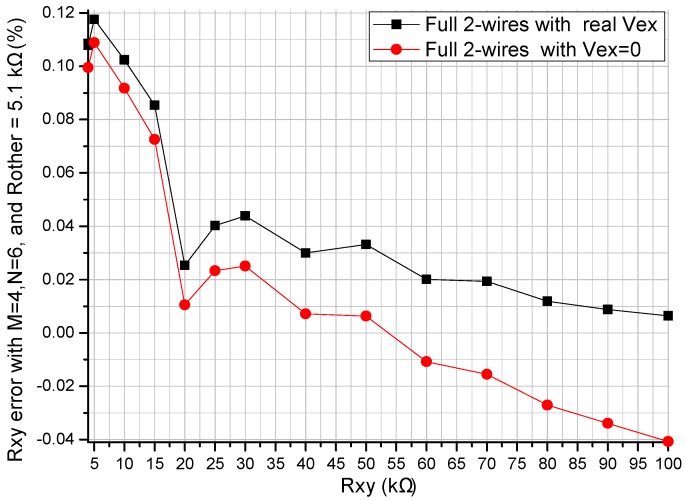
*V_ex_* effect on the EBT’s error in the full two-wire prototype circuit.

In the proposed full two-wire readout approach, the real *V_ex_* was measured and the EBT’s resistance was calculated with Equation (2). If the voltage value of the *V_ex_* was regarded as zero potential, the EBT’s resistance could also be calculated. Using the data in Figure 7, the *V_ex_* effect on the EBT’s error in the full two-wire prototype circuit is shown in Figure 8.

From the results in Figure 8, with the EBT varied from 4 kΩ to 100 kΩ, we found that the EBT’s errors in the full two-wire fast readout prototype circuit were small (<0.12%); both curves showed an obvious decline, where the curve with the *V_ex_* at zero potential showed a more obvious decline. With the EBT’s resistance at a bigger value (≥60 kΩ), the proposed full two-wire readout approach using Equation (2) showed a better performance (the EBT’s error ≤ 0.02%). Therefore, the *V_ex_*, whose effect was more obvious with an EBT of a larger resistance, had a small effect on the EBT’s error in the full two-wire prototype circuit.

## 4. Discussion

From Figure 2, the sampling channels of the full two-wire readout circuit were double the amount of the sampling channels of the one-wire readout circuit and those of the part two-wire readout circuit. Therefore, the full two-wire readout circuit was not applicable for the resistive sensor array with a large number of sampling electrodes. From the results in Figure 6, the column number had a negligible effect on the *R_xy_* errors of the full two-wire readout circuit. Thus, the full two-wire readout circuit was applicable for the resistive sensor array with a large number of driving electrodes.

From the results in Figure 3 and Figure 4, both the row resistance and the column resistance affected the *R_xy_* errors of the one-wire readout circuit with the simplest structure; with the increase of the row resistance and the column resistance, the *R_xy_* errors of the one-wire readout circuit increased. With a large row resistance, the part two-wire readout circuit with a relatively simple structure had large *R_xy_* errors even if the column resistance was small. With the increase of the resistance of *R_xy_*, both the one-wire readout circuit and the part two-wire readout circuit showed *R_xy_* errors with negative coefficients, which were more significant in the part two-wire readout circuit with a larger row resistance. The *R_xy_* errors of the full two-wire readout circuit with the most complex structure were small enough to be negligible (less than 0.1%) even if both the column resistance and the row resistance were large. From the results in Figure 5, when the column resistance and the row resistance were large, the non-scanned elements had an obvious effect on both the *R_xy_* errors of the one-wire readout circuit and those of the part two-wire readout circuit, but the non-scanned elements had almost no effect on the *R_xy_* errors of the full two-wire readout circuit.

From Figure 4 and Figure 5, with a small row resistance, the non-scanned elements and the column resistance had almost no effect on the *R_xy_* errors of the part two-wire readout circuit, and similar results were also presented in [14]. However, with a large row resistance, both the non-scanned elements and the column resistance affected the measurement accuracy of the part two-wire readout circuit. Thus, the part two-wire readout circuit had a good performance with a small row resistance. However, cable resistances existed in the row wires and the column wires, and the part two-wire readout circuit was not applicable for suppressing the crosstalk caused by long cables.

From Figure 3, Figure 4 and Figure 5, we found that the performance of the part two-wire readout circuit was worse than that of the one-wire readout circuit when the row resistance was large. As for the reason, we found in Figure 1 that all non-scanned driving electrodes of the one-wire readout circuit were connected to the zero potential while the non-scanned driving electrodes of the part two-wire readout circuit had uncertain floating potentials, which could break its ideal working condition. By connecting all non-scanned driving electrodes to the zero potential, it was possible for the part two-wire readout circuit to have a better performance even if the row resistance was large. From Figure 2, the non-scanned driving electrodes of the full two-wire readout circuit were connected to the zero potential, which guaranteed its ideal working condition based on ZPM. Also, the voltage bias caused by the row resistance was measured with an additional sampling channel and it was used to calculate the precise resistance of the EBT. Thus, the full two-wire readout circuit had the best accuracy in the three circuits.

From Figure 7 and Figure 8, the full two-wire fast readout prototype circuit had a good performance in a wide range of the elements being tested. However, from the results in Figure 8, there existed some particular points, for example at 20 kΩ of the EBT’s resistance, which might be caused by the non-linearity of the op-amp in the prototype circuit.

For quickly sensing external touch, it was necessary for resistive sensor arrays of the robots to have a readout approach with a high readout rate and good accuracy. Based on the ZPM with many synchronous sampling channels, the one-wire readout circuit and the part two-wire readout circuit were used to access all elements on one column in the array at the same time and fast readout rates, for example 1.2 kHz, were obtained [6,13]. A similar ZPM-based readout approach with a high read rate was also used in the proposed full two-wire readout circuit.

The effects of the zero potential op-amp’s gain on the readout circuits of resistive sensor arrays were analyzed in [20,23]. With a higher frequency response and a higher readout rate, the dynamic gain of the op-amp was smaller, which would degrade the measurement accuracy of the full two-wire readout circuit. For the same readout rate, there was a lower channel-switching frequency in the full two-wire readout circuit with more synchronous sampling channels than those in the readout circuits with only one sampling channel; then, a larger dynamic gain and a better accuracy could be obtained with the full two-wire readout circuit. However, one op-amp in negative feedback for every sampling electrode and every driving electrode was used in the full two-wire readout circuit and there was a risk of oscillation. By testing and obtaining the free-running oscillation frequency of the full two-wire readout circuit, special selection of the readout rate of the readout circuits could be used to avoid the risk of oscillation.

As stated above, many factors including the non-scanned elements, the driving electrodes’ number, the multiplexers’ switch-on resistances, and wire resistances and contact resistances of the long cables had less effect on the *R_xy_* errors of the full two-wire readout circuit. Using the full two-wire readout circuit, more choices, including a larger number of driving electrodes, more types of multiplexers with larger switch-on resistances, and longer cables with larger wire resistances and contact resistances, could be realized in the resistive sensor array. Thus, the full two-wire readout circuit with a fast readout rate and good measurement accuracy is useful for many applications such as flexible electronic skin, tactile sensing applications, smart clothes, *etc.* However, in the *M* × *N* resistive sensor array, many conditions including 2(*M* + *N*) wires, (*M* + *N*) op-amps, and 2*M* synchronous sampling channels are necessary for the proposed circuit. Therefore, the proposed circuit has a larger cost, a larger power consumption, and a more complex structure than the one-wire readout circuit and the part two-wire readout circuit. With a small row resistance, including the wire resistance and the contact resistance, in the resistive sensor array, the part two-wire readout circuit with a low cost and simple structure also has a fast readout rate and good measurement accuracy.

It should be noted that all analyses and results were right under the assumption that the column op-amps had sufficient driving ability and the row op-amps had very big input impedances on the inverting inputs. If the row op-amp did not have a very big input impedance or the elements in the resistive sensor array had very big resistance values, the current on the EBT would not be equal to the current on its feedback resistance. Thus, the ideal work condition was destroyed in the proposed full two-wire readout circuit and the EBT’s measurement error would be significant.

## 5. Conclusions

We presented the full two-wire approach with a high readout rate for solving the cable crosstalk problem in resistive tactile sensors. The fast readout approach provided better measurement accuracy for tactile resistive sensor arrays with long cables, which was a problem for other techniques. We verified the circuit’s performance using simulations and experiments, and we discussed the limit of the proposed full two-wire approach.

## Figures and Tables

**Table 1:** Comparison of different readout approaches of the 2D resistive sensor array.

Literature	Approaches	Readout Rate	Error Source		
			Multiplexer’s *R_sc_*	Bypass of *R_non-scanned_*	Cable Crosstalk
[4,18,19]	one-wire VFM	slow	Yes	Yes	Yes
[7,15]	one-wire ZPM	slow	Yes	Yes	Yes
[16]	two-wire VFM	slow	No	No	No
[17]	two-wire S-NSDE-EP method	slow	No	No	No
[1,2]	passive integrator method	fast	No	No	Yes
[12]	resistance matrix approach	fast	No	Yes	Yes
[5,6,13,20]	Multi-channel one-wire ZPM	fast	Yes	Yes	Yes
[14]	Multi-channel part two-wire ZPM	fast	No	No	Partly
Proposed	Multi-channel full two-wire ZPM	fast	No	No	No
